# Oromaxillary Prosthetic Rehabilitation of a Maxillectomy Patient Using a Magnet Retained Two-Piece Hollow Bulb Definitive Obturator; A Clinical Report

**DOI:** 10.1155/2013/190180

**Published:** 2013-03-04

**Authors:** Jafar Abdulla Mohamed Usman, Anuroopa Ayappan, Dhanraj Ganapathy, Nilofer Nisha Nasir

**Affiliations:** ^1^Subsitutive Dental Sciences, College of Dentistry, King Khaled University, P.O. Box 3263, Abha 61471, Saudi Arabia; ^2^Department of Prosthodontics, Sree Mookambika Institute of Dental Sciences, Kulashekaram, India; ^3^Derartment of Prosthodontics, Saveetha Dental College, Chennai, India; ^4^Department of Conservative Dentistry and Endodontics, Rajah Mutthiah Dental College, Annamalai University, Chidambaram, Tamilnadu, India

## Abstract

Resection of a malignant lesion involving the maxilla produces severe oromaxillary defect that can seriously jeopardize the normal phonetics of the patient. These defects are effectively managed by well-designed and fabricated obturator. This paper discusses the oromaxillary prosthetic rehabilitation of a maxillectomy patient using a magnet retained two-piece hollow bulb definitive obturator.

## 1. Introduction

Palatal defects impart significant physical and psychological damage to the ailing patient. The various etiological factors constituting these defects can be segregated into two broad categories, namely, the congenital and the acquired defects. The acquired defects could be due to trauma, infection, and iatrogenic as a result of surgical resection of malignant as well as nonmalignant lesions. The oromaxillary defect causes transportation of oral and nasal microflora, regurgitation of oral fluids, alteration in voice due to asynchrony in resonance, and difficulty in speech as well as swallowing. Hence effective treatment modalities to treat these defects become mandatory as a clinical protocol.

## 2. Case Report

A male patient aged fifty years was referred to the hospital with the history of swelling in left maxillary posterior region and mobility of teeth from second premolar to third molar. The patient was examined both clinically and radiographically. The lesion was sent for biopsy and histodiagnosed as squamous cell carcinoma involving the left maxillary antrum. The patient underwent a total maxillectomy of the left maxilla along with block dissection of lymph nodes in the neck. After surgery, the patient was rehabilitated with an interim obturator for a month and surgical site was allowed to heal. The patient reported after a month to the department of prosthodontics with complaints of change in voice, regurgitation of fluids to nose, and burning sensation in the mouth and nose.

**Figure 1:**
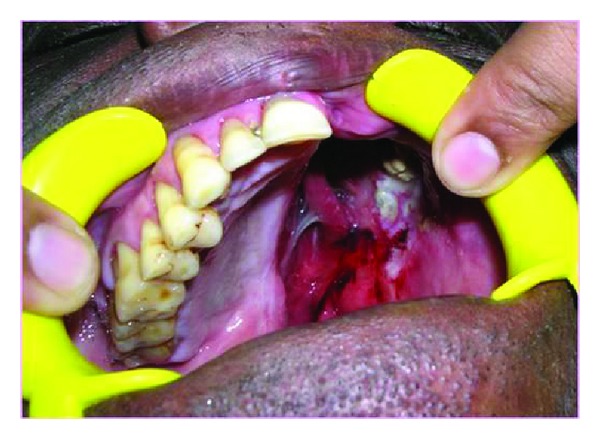
Maxillectomy defect.

**Figure 2:**
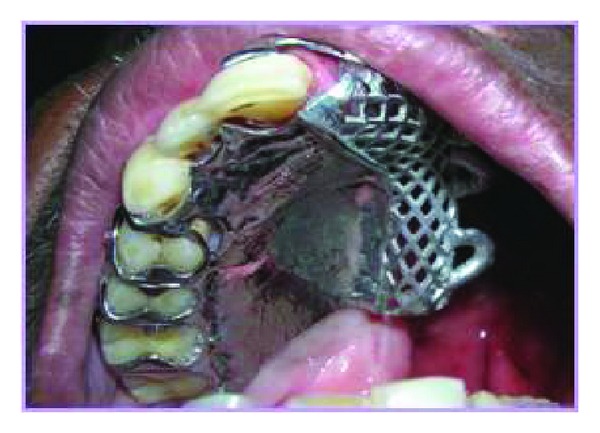
Framework try-in.

On clinical examination, a defect due to maxillectomy was present from the midline to the soft palate on the left side. The tissue showed good signs of healing and the defect was classified as Aramany class two defect which measured 2 cm mediolaterally and 3.5 cm superoinferiorly (Figure 1) [1]. The remaining teeth exhibited significant periodontal breakdown and mild supraeruption. The treatment plan was ruled out and a definitive prosthesis was decided to be given to the patient. The defect was packed with gauze so as to prevent the ingress of the impression material into the nasal cavity. Primary impression was made with irreversible hydrocolloid (Zelgan 2000) and casts were obtained. The casts were surveyed with Jelenko dental surveyor and the undercuts were established and direct retainers in the form of embrasure clasps were planned in teeth numbers 15, 16, 17, and 18 [2]. Complete coverage palatal maxillary major connector with chrome cobalt alloy and mesh type denture base minor connector were selected (Figure 2).

**Figure 3:**
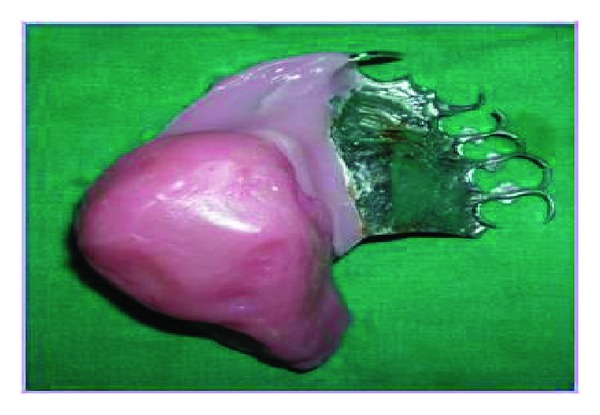
Completed prosthesis.

**Figure 4:**
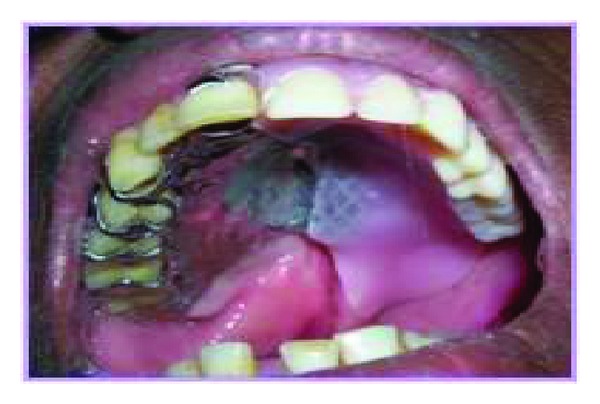
Insertion of the prosthesis.

Careful surveying revealed the defect and the palatal plate major connector had varied paths of insertion. Hence a two-piece hollow bulb obturator was planned for treating the patient. Special trays were constructed with suitable tissue stops from the primary cast. The secondary impressions were secured by twin stage impression procedure where the impression of the defect was made with putty consistency polyvinyl siloxane (Aquasil) and was picked up by a full arch impression with medium consistency polyvinyl siloxane and poured with type IV stone (ultra rock) and a secondary cast was obtained. The lateral undercuts in the defective area were blocked out and a hollow bulb obturator was processed using a lost salt technique. The hollow bulb obturator was tried in patient's mouth for retention and comfort. Then the cast partial denture framework with the prosthetic teeth was tried in the patient's mouth and evaluated for extension, retention, stability, occlusion, and phonetics (Figure 3). Cobalt samarium magnets of 4 mm dimension were placed over the tissue side of cast partial denture framework and the corresponding pair was fixed on the obturator using autopolymerising acrylic resin (DPI, India). The retention was excellent with magnetic keepers. The obturator was subsequently relined with permanent soft liner (Perma Soft) to completely obturate the lateral defects. This was tried in the patient (Figure 4). The patient was reviewed periodically for 12 months. The patient experienced great comfort, enhanced mastication, and phonetics with the prosthesis.

## 3. Discussion

Acquired maxillary defects due to various etiologic factors pose a great challenge to the clinician. The patient suffers a severe deficit in the masticatory and phonetics function. Salvaging teeth during the surgical procedure reduces the number of occlusal units in the oral cavity and significantly hampers masticatory efficiency [3]. It also substantially compromises pronunciation of words which occurs in the form of nasal twang and increased cubicle space resulting in poor articulation with linguodental and linguopalatal consonants [4]. One of the serious dysfunction caused by acquired palatomaxillary defect is the intertransportation of micro organisms between the oral and nasal cavity. The nasal cavity is lined by pseudo stratified ciliated columnar epithelium and goblet cells present there aggressively attract oral flora [5]. In addition, regurgitation and transportation of food and fluids from the oral cavity to nasal cavity via the defect cause severe discomfort to the patient.

The obturator prosthesis is designed to seal the defect, functions efficiently as it prevents the infiltration of food, fluids, and flora from the oral to nasal chambers and vice versa. It tremendously improves the quality of voice as it completely seals the lateral palatal defect as well as the maxillary defect. One of the problems associated with oromaxillary obturators is insertion of the prosthesis due to compromised anatomic morphology in different planes [6]. Hence it is mandatory to design an obturator in two sections wherein the obturator is inserted initially followed by oropalatal metal framework [7]. The two sections are retained together in function as one unit by retentive devices subsequently.

There are several retentive devices available to secure the two sections in position [8]. Among the various retentive devices magnetic attachments are more user friendly and cost effective when compared to internal attachments which required extreme precision and good neuromuscular coordination from the patient to insert and use the prosthesis. When compared to conventional iron boron magnets, cobalt samarium magnets undergo less corrosion and hence they were selected for this case. The disadvantage of magnetic attachment is the possible loss of magnetism during function during extended period of time. But they can be magnetized with reasonable ease and can be induced to function immediately [9].

Another problem with maxillofacial obturator is the increased weight of the prosthesis due to the bulk of the resin occupied in the defect area and hence the weight was reduced by fabrication of hollow bulb obturator using lost salt technique [10]. The palatal obturator in the defect which is subsequently relined with a soft liner greatly enhances the comfort of the patient as it is flexible and protects the integrity of the adjoining moving tissues. A proper maintenance regimen with chlorhexidine mouth wash and a comprehensive education on the manipulation of the prosthesis increases the success and survival rate of oromaxillary obturator.

## 4. Conclusion

This paper discussed the prosthetic management of acquired Oromaxillary defect with a two-piece cast partial hollow bulb definitive obturator with magnetic attachment and tissue liners.
